# Disparities in Unintentional Occupational Injury Mortality between High-Income Countries and Low- and Middle-Income Countries: 1990–2016

**DOI:** 10.3390/ijerph15102296

**Published:** 2018-10-19

**Authors:** Yue Wu, David C. Schwebel, Guoqing Hu

**Affiliations:** 1 Department of Environmental and Occupational Health, Xiangya School of Public Health, Central South University, 110 Xiangya Rd., Changsha 410078, China; wuyue7802@csu.edu.cn; 2 Department of Psychology, University of Alabama at Birmingham, Birmingham, AL 35294, USA; schwebel@uab.edu; 3 Department of Epidemiology and Health Statistics, Xiangya School of Public Health, Central South University, 110 Xiangya Rd., Changsha 410078, China

**Keywords:** occupational injury, unintentional, disparity

## Abstract

Objective: Using estimates from the Global Burden of Disease (GBD) study, we examined differences in unintentional occupational injury mortality rates from 1990 to 2016 between high-income countries (HICs) and low- and middle-income countries (LMICs). Methods: Unintentional occupational injury mortality rates were obtained through the GBD online visualization tool. We quantified mortality changes over time for common external causes of injury for ages 15–49 years and 50–69 years separately in HICs and LMICs using negative binomial regression models. Results: In 2016, there were 24,396 and 303,999 unintentional occupational injury deaths among individuals aged 15 to 69 years in HICs and LMICs, respectively, corresponding to 3.1 and 7.0 per 100,000 people. Between 1990 and 2016, unintentional occupational injury mortality for people aged 15–69 years dropped 46% (from 5.7 to 3.1 per 100,000 people) in HICs and 42% in LMICs (from 13.2 to 7.0 per 100,000 people). Sustained and large disparities existed between HICs and LMICs for both sexes and both age groups during 1990–2016 (mortality rate ratio: 2.2–2.4). All unintentional occupational injury causes of death displayed significant reduction with one exception (ages 15–49 years in HICs). Country-specific analysis revealed large variations in unintentional occupational injury mortality and changes in occupational injury mortality between 1990 and 2016. Conclusions: Despite substantial decreases in mortality between 1990 and 2016 for both HICs and LMICs, a large disparity continues to exist between HICs and LMICs. Multifaceted efforts are needed to reduce the disparity.

## 1. Introduction

The Sustainable Development Goals (SDGs) include a goal for “Decent Work” and a target to focus on protecting labor rights and promoting safe and secure working environments for all workers [1]. Prevention of occupational injury forms a central component of a “decent work” environment, and it is widely recognized that no country demonstrates a high level of competitiveness and productivity while maintaining poor safety records for its workers [2].

Previous research has estimated occupational injury mortality at national [3,4,5,6,7,8], regional [9,10], and global levels [11,12,13,14,15,16,17,18,19]. In select countries, most of them high-income, data are generally considered to be of high quality [20] and indicate that occupational injury mortality varies across high-income countries (HICs), ranging from 3.6 per 100,000 full-time equivalent (FTE) workers in the United States [3] to 9.6 in South Korea [5]. Data from low- and middle-income countries (LMICs) are more sparse and of varying quality [7,8]. However, some estimates are available. Jin estimated a mortality rate of 9.3 per 100,000 workers in China, for example [7], and Abas and colleagues reported an average annual mortality of fatal occupational injuries of 9.2 per 100,000 workers in Malaysia from 2002–2006 [8].

Estimates from certain global regions are published also. Giuffrida estimated about 27,270 fatal occupational injuries occurred in Latin America and the Caribbean [10]. The European Union (EU) reported 3739 deaths from occupational injuries in the EU in 2014, equaling about 2.3 fatal injuries per 100,000 employed persons [9].

Globally, fatal occupational injury numbers and rates have been estimated discretely [11,12,13,14,15,16,17,18], with statistical models changing across multiple rounds of estimation. Hämäläinen and colleagues, for example, recently reported an 8% increase in global occupational injury deaths between 2010 and 2014 [18]. Concha-Barrientos et al estimated an annual fatal unintentional occupational injury deaths of 312,000 in the world in 2000 [19].

Previous studies have not assessed change in global occupational injury mortality over long time periods, limiting our ability to examine how changes in occupational safety and health (OSH) policies may have influenced injury rates [14]. Disparities across country income level (i.e., HICs versus LMICs) over time are unexamined and critical to inform progress toward Sustainable Development Goals (SDGs) for “Decent Work” [1].

This study was designed therefore to offer a comprehensive global view of the scope of the public health challenge of unintentional occupational injury deaths, as well as trends over time in the rate of unintentional occupational injury mortality in 194 countries and territories worldwide. We considered rates in both low and middle-income and high-income countries, and we considered trends over time as well as current fatal occupational injury rates.

## 2. Methods

### 2.1. Data Source

We retrieved all data through the global burden of disease (GBD) online visualization tool developed by the GBD Study group [21]. The dataset quantifies the comparative magnitude of health loss from diseases, injuries, and risks by location (national and in some cases subnational), age, sex, cause, and risk factor over time [22]. The GBD 2016 provides the estimates of burden attributed to 86 risk factors and their corresponding 95% uncertain interval (95% UI) based on GBD models and multiple raw data sources, including vital registration systems, verbal autopsy, cancer registry, police records, sibling history, surveillance, and survey/census data [23].

GBD 2016 uses the population attributable fraction (PAF) to estimate the number of injury deaths attributable to occupational risk for a given age, sex, location, and year [23], rather than directly obtaining occupational injury deaths from classification of death as many other studies do. The PAF represents the proportion of outcome that would be reduced in a given year if the exposure to a risk factor in the past were reduced to the counterfactual level of the theoretical minimum risk exposure level. GBD attribution methods are detailed elsewhere [23]. The GBD study group defines occupational injuries as injury events related to work or through the individual’s occupational duties but excludes fatalities occurring during commutes to and from work [23], and classifies them using the 10th International Classification of Diseases (ICD-10) codes [24]. To satisfy other research purposes, the GBD study group does not provide the burden estimates of unintentional occupational injury for the economically active population (18–69 years) [25], but instead extends the age of analysis down to 15 years old. Thus, we considered the results for the age group 15–69 years as the working population to calculate unintentional occupational injury burden. 

The attribution results of eight major causes of unintentional occupational injuries are presented, consisting of: (1) road injury (ICD-10 codes: V01–V04.99, V06–V80.929, V82–V82.9, V87.2–V87.3); (2) other transport injury (ICD-10 codes: V00–V00.898, V05–V05.99, V81–V81.9, V83–V86.99, V88.2–V88.3, V90–V98.8); (3) falls (ICD-10 codes: W00–W19.9); (4) drowning (ICD-10 codes: W65–W70.9, W73–W74.9); (5) poisonings (ICD-10 codes: X46–X47, X47.1–X47.8, X48–X48.9); (6) exposure to mechanical forces (ICD-10 codes: W20–W38.9, W40–W43.9, W45.0–W45.2, W46–W46.2, W49–W52, W75–W75.9); (7) foreign body (ICD-10 codes: W44–W45, W45.3–W45.9, W78–W80.9, W83–W84.9); and (8) other injuries (ICD-10 codes: W32–W34.9, W39–W39.9, W52.0–W62.9, W64–W64.9, W75–W75.9, W77–W77.9, W81–W81.9, W85 –W94.9, W97.9, W99–W99.9, X00–X06.9, X08–X32.9, X39–X39.9, X50–X54.9, X57–Y84.9, Y88–Y88.3) [24].

### 2.2. Data Analysis

GBD 2016 offers outcome estimates for 194 countries, which are divided into 56 HICs and 138 LMICs according to the World Bank’s classification [26]. Note that the term “country” does not imply political independence, but refers to any territory for which authorities report separate social or economic statistics. 

We analyzed the data in three steps. First, we quantified unintentional occupational injury mortality differences over time between HICs and LMICs, both overall and by sex (male vs. female) and age (ages 15–49 vs. ages 50–69), respectively. We divided the analysis into two age groups given both variations in physiological condition and in exposure to certain occupational risks for younger and older adults [27]. Second, we conducted similar analyses quantifying injury mortality between HICs and LMICs by cause and age group. Third, we reported country-specific mortality changes between 1990 and 2016 by age group and country income (HICs versus LMICs) separately.

Percent change in mortality rates and its 95% confidence intervals (CI) were estimated via negative binomial regression to quantify mortality changes between 1990 and 2016, which was calculated as “(mortality rate ratio: 1) × 100”. All statistical analyses were completed using Stata version 12.1 (StataCorp LLC, College Station, TX, USA).

## 3. Results

**Figure 1:**
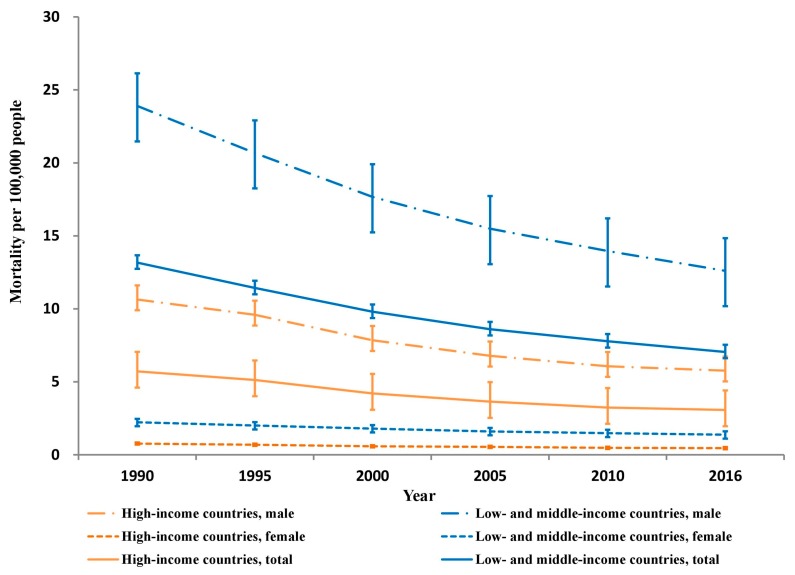
Unintentional occupational injury mortality per 100,000 persons by country income and sex, 1990–2016.

Between 1990 and 2016, the estimated crude number of deaths from unintentional occupational injury dropped from 37,581 to 24,396 in HICs and from 355,812 to 303,999 in LMICs for the working age group of 15 to 69 years. These drops correspond to age-specific mortality decreases of 46% in HICs (from 5.7 to 3.1 per 100,000 people; 95% CI: 45–47%) and 43% in LMICs (from 13.2 to 7.0 per 100,000 people; 95% CI: 42–44%) (Figure 1). 

**Figure 2:**
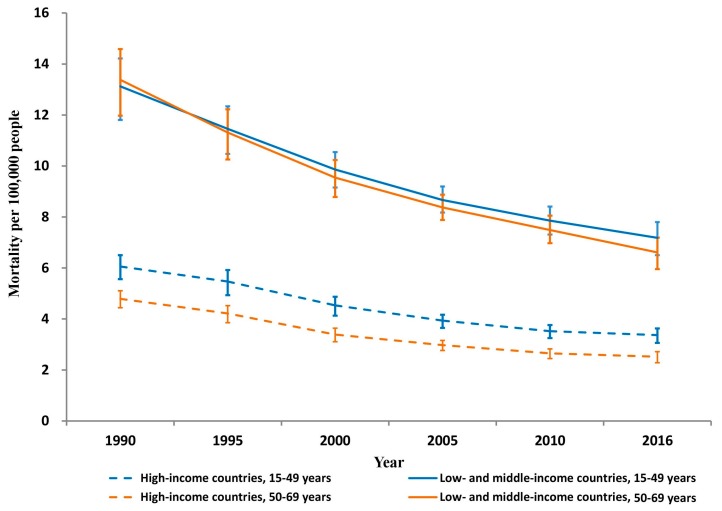
Unintentional occupational injury mortality per 100,000 persons by country income and age group, 1990–2016.

Similar substantial reductions occurred in both sexes in HICs and LMICs (males: 47% in HICs and 47% in LMICs; and females: 40% in HICs and 38% in LMICs). From 1990 to 2016, both age groups had much higher unintentional occupational injury mortality rates in LMICs than in HICs (mortality rate ratio: 2.1–2.2 in 15–49 years and 2.6–2.8 in 50–69 years) (Figure 2). 

Substantial mortality decreases occurred between 1990 and 2016 in both HICs and LMICs for 15–49-year-olds (44% and 45%, respectively) and for 50–69-year-olds (47% and 51%, respectively) (Table 1 and Figure 2). 

Subgroup analysis by cause, age group and country income showed large mortality differences between HICs and LMICs (mortality rate ratio: 1.4–4.8 for 15–49 years and 1.4–5.4 for 50–69 years in 1990; 1.1–4.4 for 15–49 years and 1.0–4.2 for 50–69 years in 2016) (Table 1). Consistent, large or moderate decreases occurred in all subgroup unintentional occupational injury mortality rates (from −70% to −28%) except for mortality from foreign body in age group 15–49 years for HICs (−11%) between 1990 and 2016 (Table 1). In addition, country-specific analysis showed great variations in unintentional occupational mortality in 1990 and in 2016, and in mortality changes between 1990 and 2016 for both age groups within both HICs and LMICs.

**Figure 3:**
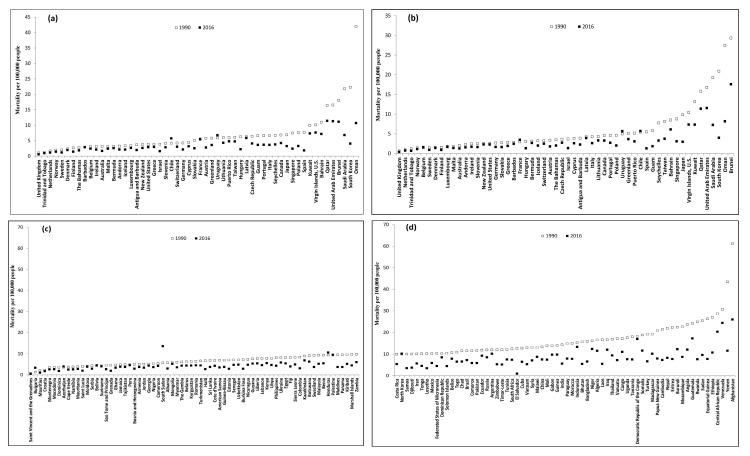

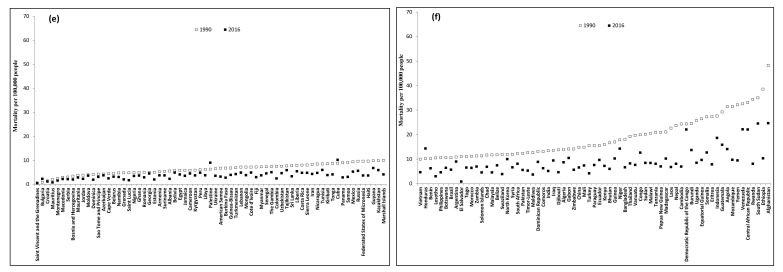
Country-specific unintentional occupational injury mortality of 196 countries between 1990 and 2016. (**a**) 15–49 years in HICs; (**b**) 50–69 years in HICs; (**c**) 15–49 years in LMICs; (**d**) 15–49 years in LMICs (continued); (**e**) 50–69 years in LMICs; and (**f**) 50–69 years in LMICs (continued).

For HICs, the mortality gap between the highest and lowest-risk countries was 41 times in 1990 (1.0 per 100,000 persons in United Kingdom and 42.0 per 100,000 persons in Oman) and 57 times in 2016 (0.2 per 100,000 persons in Denmark and 11.3 per 100,000 persons in United Arab Emirates) for age group 15–49 years (Figure 3a, Table S1) and 42 times in 1990 (0.7 per 100,000 persons in United Kingdom and 29.3 per 100,000 persons in Brunei) and 44 times in 2016 (0.4 per 100,000 persons in United Kingdom and 17.6 per 100,000 persons in Brunei) for age group 50–69 years (Figure 3b, Table S3). The largest mortality decrease between 1990 and 2016 were −82% (95% CI: −83% to −81%) and −81% (95% CI: −83% to −79%) in South Korea for age group 15–49 years and 50–69 years, respectively, compared to a 37% (95% CI: 18% to 57%) increase in Chile and 17% (95% CI: 2% to 34%) increase in France for the two age groups.

For LMICs, the mortality gap between the highest-and lowest-risk countries was 121 times in 1990 (0.5 per 100,000 persons in Saint Vincent and the Grenadines and 61.2 per 100,000 persons in Afghanistan) and 52 times in 2016 (0.5 per 100,000 persons in Saint Vincent and the Grenadines and 26.0 per 100,000 persons in Afghanistan) for age group 15–49 years (Figure 3c,d, Table S2) and 80 times in 1990 (0.6 per 100,000 persons in Saint Vincent and the Grenadines and 48.2 per 100,000 persons in Afghanistan) and 62 times in 2016 (0.4 per 100,000 persons in Saint Vincent and the Grenadines and 24.7 per 100,000 persons in Afghanistan) for the age group 50–69 years (Figure 3e,f, Table S4). The most notable LMIC mortality decrease between 1990 and 2016 occurred in El Salvador for age groups 15–49 years (-93%, 95% CI: −95% to −89%) and for 50–69 years (-92%, 95% CI: -96% to −84%), compared to increases of 155% (95% CI: 84% to 252%) and 169% (95% CI: 56% to 364%) for the two age groups in Bulgaria. 

## 4. Discussion

Our findings indicate that unintentional occupational injury continues to account for substantial numbers of deaths globally. We estimate 328,035 persons aged 15 to 69 years died in 2016 from unintentional occupational injury, slightly higher than 312,000 deaths estimated for 2000 by Concha-Barrientos [19] and slightly lower than Hämäläinen’s estimate of 380,500 for 2014 [18].

Our results show over 92% of unintentional occupational injury deaths occurred in LMICs in 2016. There exists a great disparity in unintentional occupational injury mortality between HICs and LMICs (3.1 vs. 7.0 per 100,000 people). Disparities in unintentional occupational injury mortality between HICs and LMICs persisted throughout our study period. This finding underscores the urgent public health need for leadership and teamwork to reduce unintentional occupational mortality rates in LMICs. The proverbial “low-hanging fruit” to accomplish this may be to adopt strategies used in HICs to improve occupational health and safety in LMICs. As an example, a recent survey illustrates the gap in the percentage of workers with access to occupational health services (OHS) between LMICs and HICs [28]; coverage is generally low in LMICs like Brazil (26%), China (10%), and India (<10%), which offer coverage to very few workers, whereas coverage in HICs general hovers at or above 75% of workers (e.g., in France, Italy, and Japan). 

LMICs also have been slow to ratify occupational safety and health (OSH) related conventions. For example, ratification figures for LMICs in Asia, which represent 21% of all ILO member states, show only 7% of the global OSH conventions were ratified [2]. Failure to ratify OSH conventions in Asia may be politically motivated to some extent, but it may also represent insufficient infrastructure to implement policies. Through the natural processes of globalization and economic development, many hazardous and labor-intensive activities like manufacturing have moved from HICs to LMICs, especially in Asia, where labor costs less and is readily available [29]. Agricultural distribution has also globalized, leading to increases in agricultural work in LMIC where laborers may face substantial hazards.

In our examination of unintentional occupational injury mortality rates over time, we detected significant decreases globally. Economic growth accompanied by greater investment in safety technologies and improved working conditions could explain decreases in fatal occupational injuries in many countries over time [30,31]. The declines in LMICs were similar to those in HICs, although the cause of the declines in the two regions may be distinct. Decreases in HICs might be attributed to a combination of two factors. First, many countries have implemented stricter legislation relating to occupational safety. As an example, new member states joining the EU, such as Czech Republic, Hungary, and Poland [14,32], have adopted EU policies that greatly improved their worker’s safety. Second, injury prevention efforts have been implemented broadly and documented in HICs to improve work conditions and attention to safety. For example, the United States Occupational Safety and Health Administration (OSHA) has brought about significant safety regulations, mandatory workplace safety controls, and worker training programs over the past decades in the United States [33]. 

In LMICs, the decrease in unintentional occupational injury deaths may be attributed to shifts in patterns of employment. As economies develop, workers move from more dangerous occupations (e.g., production, construction, mining, farming) to safer service industries. In most HICs, over 2/3 of workers are now employed in service occupations [29], and similar trends are occurring globally [34]. Over the past decade, many more countries, including many LMICs, have recognized the importance of OSH, and the urgency to prioritize injury prevention strategies at workplaces. OSH education and training facilities have expanded in many countries [13] and could serve as part of a broader effort to promote occupational safety and health in the world.

One other finding of note was the fact that we discovered substantial disparities in unintentional occupational injury mortality rates and change patterns over time within the HIC and LMIC groupings. These differences may reflect the effect of variations in types of industries, occupational activities, employment characteristics, economic growth or stagnation, and implementation of safety policies/measures across countries. As an example, the impact of the recent global recession on occupational safety and health varied among countries, with high-income European nations experiencing changes in investment portfolios in 2008 that spanned a wide range, from −30.6% in Ireland to −3.2% in Denmark [35]. In response to negative economic situations, many governments curtailed resources allocated to labour administration systems and labour safety initiatives, which may have impacted occupational injury rates [35]. Some countries did not curtail such spending, however. Italy, for example, is documented to have increased occupational health and safety spending by 21% from 2006 to 2013 [36]. Another possible factor in all countries is the impact of climate change. Previous studies indicate that extreme weather events such as high ambient temperature may impact occupational injury rates in HICs [37,38,39]. 

Within country groupings of similar economic status that have disparities in unintentional occupational injury rates, communication and cooperation may be fruitful to facilitate efforts that promote safety in countries with poor performance in unintentional occupational injury prevention. Globally harmonized actions could be initiated by international organizations such as the United Nations and World Health Organization to reduce disparities.

## 5. Limitations

This study was primarily limited by the details and quality of the GBD risk-attribution estimates. For example, the GBD estimates rely on sparse national data in some regions, and generally assume that relative risks are uniform across neighboring and similar countries for a given age-sex group [23]. While the GBD study group uses a standardized protocol to increase data comparability across countries and regions, some issues like changes in data reporting for specific countries are difficult to adjust accurately because relevant details about data reporting are not always available or shared with the GBD study group by government authorities. Further, the lack of coverage of self-employed workers and the sparsity of data from small-sized enterprises may lead to bias in fatal occupational injury estimates. Model-based attribution analyses may also bias estimates of occupational injury mortality since they rely highly on model assumptions rather than actual data from occupation injury surveillance. 

We relied on 2016 economic data to classify countries. These data are different from 1990 economic status data, of course, and may somewhat impact the interpretation of results. Finally, we used only data from 1990 and from 2016 to measure mortality changes, which overlooks possible fluctuations in occupational injury mortality rates during the intervening years.

## 6. Conclusions

Despite substantial decreases in unintentional occupational injury mortality between 1990 and 2016 for both HICs and LMICs, a large disparity exists between HICs and LMICs. Efforts should be taken to diminish the disparity through policy and behavioral measures that reduce occupational injury risk to workers worldwide.

## Figures and Tables

**Table 1:** Age-specific unintentional occupational injury mortality (/100,000 persons) by country income, age group, and cause among 15 to 69 years between 1990 and 2016.

Country Income/Age Group/Cause	1990		2016		Percent Change in Rate (95% CI)
	Deaths	Rate	Deaths	Rate	
HICs, 15–49 years					
All injury	29,113	6.0	17,462	3.4	−44 (−45, −43)
Road injury	18,859	3.9	10,959	2.1	−46 (−47, −44)
Other transport injury	1227	0.3	894	0.2	−32 (−37, −26)
Falls	2343	0.5	1794	0.3	−28 (−33, −24)
Drowning	1774	0.4	1089	0.2	−43 (−47, −38)
Poisonings	703	0.1	331	0.1	−56 (−61, −50)
Exposure to mechanical forces	1881	0.4	979	0.2	−51 (−55, −47)
Foreign body	638	0.1	606	0.1	−11 (−20, −1)
Other injuries	1688	0.3	810	0.2	−55 (−59, −51)
LMICs, 15–49 years					
All injury	288,906	13.1	237,333	7.2	−45 (−45, −45)
Road injury	151,573	6.9	139,998	4.2	−38 (−39, −38)
Other transport injury	11,286	0.5	9720	0.3	−43 (−44, −41)
Falls	25,668	1.2	21,716	0.7	−44 (−45, −43)
Drowning	32,173	1.5	20,129	0.6	−58 (−59, −58)
Poisonings	7906	0.4	4353	0.1	−63 (−65, −62)
Exposure to mechanical forces	19,697	0.9	14,607	0.4	−51 (−52, −49)
Foreign body	4141	0.2	4177	0.1	−33 (−36, −30)
Other injuries	36,462	1.7	22,633	0.7	−59 (−59, −58)
HICs, 50–69 years					
All injury	8468	4.8	6934	2.5	−47 (−49, −46)
Road injury	4155	2.4	3074	1.1	−52 (−55, −50)
Other transport injury	342	0.2	345	0.1	−35 (−44, −25)
Falls	1608	0.9	1631	0.6	−35 (−39, −30)
Drowning	500	0.3	470	0.2	−40 (−47, −32)
Poisonings	197	0.1	109	0.0	−65 (−72, −55)
Exposure to mechanical forces	669	0.4	431	0.2	−59 (−63, −53)
Foreign body	428	0.2	455	0.2	−32 (−40, −22)
Other injuries	569	0.3	419	0.2	−53 (−58, −46)
LMICs, 50–69 years					
All injury	67,203	13.4	66,666	6.6	−51 (−51, −50)
Road injury	30,299	6.0	34,553	3.4	−43 (−44, −42)
Other transport injury	2561	0.5	2496	0.2	−51 (−54, −49)
Falls	11,770	2.3	12,300	1.2	−48 (−49, −47)
Drowning	5715	1.1	4467	0.4	−61 (−63, −60)
Poisonings	2117	0.4	1261	0.1	−70 (−72, −68)
Exposure to mechanical forces	4280	0.9	3586	0.4	−58 (−60, −56)
Foreign body	1671	0.3	1629	0.2	−51 (−55, −48)
Other injuries	8790	1.7	6374	0.6	−64 (−65, −63)

95% CI: 95% confidence interval; HICs: High-income countries; and LMICs: Low- and middle-income countries.

## Supplementary Materials

The following are available online at http://www.mdpi.com/1660-4601/15/10/2296/s1, Table S1: country-specific unintentional occupational injury mortality (/100,000 persons) for age group 15–49 year in HICs between 1990 and 2016, Table S2: country-specific unintentional occupational injury mortality (/100,000 persons) for age group 15–49 years in LMICs between 1990 and 2016, Table S3: country-specific unintentional occupational injury mortality (/100,000 persons) for age group 50–69 year in HICs between 1990 and 2016, Table S4: country−specific unintentional occupational injury mortality (/100,000 persons) for age group 50−69 year in LMICs between 1990 and 2016.
