# Supplementary material for: Disparities in Unintentional Occupational Injury Mortality between High-Income Countries and Low- and Middle-Income Countries: 1990–2016
Source: Int J Environ Res Public Health. 2018 Oct 19;15(10):2296. doi: 10.3390/ijerph15102296 (PMC6210857; doi:10.3390/ijerph15102296)
Supplement: Supplementary file 1 [file ijerph-15-02296-s001.pdf]

International Journal of
Environmental Research and Public Health

MDPI

Supplements:

Table S1. Country-specific unintentional occupational injury mortality (/100,000 persons) for age group 15–49 year in HICs between 1990 and 2016.

Country	1990	2016	% Change in Rates (95% CI)
United Kingdom	1.01	0.56	−44 (−54, −33)
Trinidad and Tobago	1.12	0.95	−15 (−71, 145)
Netherlands	1.64	1.14	−31 (−47, −9)
Norway	1.92	1.41	−27 (−53, 15)
Sweden	2.00	1.15	−42 (−59, −18)
Denmark	2.36	1.96	−17 (−42, 21)
Finland	2.65	1.42	−46 (−65, −19)
The Bahamas	2.71	1.84	−32 (−84, 179)
Barbados	2.89	2.87	−1 (−75, 298)
Belgium	3.04	2.41	−21 (−37, 1)
Ireland	3.09	2.11	−32 (−54, 1)
Australia	3.12	1.67	−47 (−55, −36)
Malta	3.14	2.40	−24 (−77, 159)
Bermuda	3.17	2.56	−24 (−77, 159)
Andorra	3.22	2.12	−19 (—)
Iceland	3.30	2.10	−36 (−84, 168)
Luxembourg	3.33	2.65	−21 (−72, 125)
Antigua and Barbuda	3.66	1.96	−46 (—)
New Zealand	3.78	2.63	−31 (−51, −1)
United States	3.79	2.91	−23 (−26, −20)
Greece	3.84	2.95	−23 (−38, −4)
Israel	3.85	1.59	−59 (−70, −42)
Slovenia	4.13	2.83	−32 (−58, 11)
Chile	4.20	5.73	37 (18, 57)
Switzerland	4.21	3.01	−28 (−44, −9)
Germany	4.23	2.12	−50 (−54, −45)
Cyprus	4.42	3.16	−29 (−65, 46)
Slovakia	5.04	2.51	−50 (−63, −34)
France	5.54	5.41	−2 (−9, 5)
Austria	5.77	2.76	−52 (−62, −40)
Greenland	5.81	3.58	−38 (—)
Uruguay	5.90	6.74	14 (−14, 51)
Lithuania	5.99	4.30	−28 (−48, −1)
Puerto Rico	6.00	4.78	−20 (−40, 6)
Taiwan	6.08	4.74	−22 (−30, −13)
Hungary	6.23	2.21	−65 (−72, −56)
Latvia	6.29	5.85	−7 (−35, 32)
Czech Republic	6.42	4.07	−37 (−47, −25)
Guam	6.59	3.62	−45 (−87, 128)
Portugal	6.62	3.66	−45 (−54, −34)
Italy	6.65	3.62	−46 (−50, −41)
Seychelles	6.65	3.76	−43 (−92, 286)
Canada	6.81	4.21	−38 (−44, −32)
Japan	6.86	3.22	−53 (−56, −50)
Singapore	7.46	2.39	−53 (−56, −50)

Poland	7.59	3.24	−57 (−61, −53)
Spain	7.72	1.83	−76 (−79, −74)
Kuwait	9.93	7.33	−26 (−41, −7)
Virgin Islands, U.S.	10.09	7.62	−24 (−81, 196)
Bahrain	10.97	7.15	−35 (−57, −1)
Qatar	16.38	11.4	−30 (−49, −5)
United Arab Emirates	16.52	11.28	−32 (−42, −20)
Brunei	18.03	11.11	−38 (−64, 5)
Saudi Arabia	21.82	6.81	−69 (−71, −66)
South Korea	22.28	4.03	−82 (−83, −81)
Oman	42.00	10.68	−75 (−78, −70)

95% CI: 95% confidence interval; "—": 95% CI was unstable due to extremely small numbers of deaths and population.

Table S2. Country-specific unintentional occupational injury mortality (/100,000 persons) for age group 15–49 years in LMICs between 1990 and 2016.

Country	1990	2016	% Change in Rates (95% CI)
Saint Vincent and the Grenadines	0.52	0.49	−4 (—)
Bulgaria	1.31	3.33	155 (84, 252)
Mauritius	2.07	0.99	−52 (−81, 23)
Croatia	2.21	1.72	−22 (−50, 21)
Montenegro	3.41	2.57	−25 (−70, 90)
Macedonia	3.47	2.64	−24 (−54, 24)
Dominica	3.53	1.80	−49 (—)
Azerbaijan	3.57	3.97	11 (−11, 38)
Cape Verde	3.71	2.47	−34 (−78, 104)
Namibia	3.75	2.55	−32 (−60, 14)
Mauritania	3.93	2.74	−30 (−54, 6)
Saint Lucia	4.01	1.94	−52 (—)
Moldova	4.12	3.87	−6 (−30, 27)
Serbia	4.27	2.93	−31 (−45, −14)
Bolivia	4.31	4.54	5 (−14, 30)
Suriname	4.41	4.02	−9 (−62, 117)
Sao Tome and Principe	4.49	2.63	−41 (−91, 276)
Grenada	4.53	1.91	−58 (−96, 339)
Ghana	4.64	3.33	−28 (−38, −17)
Jamaica	4.78	3.85	−20 (−44, 16)
Tajikistan	4.80	3.75	−22 (−38, −1)
Peru	4.81	4.57	−5 (−15, 6)
Bosnia and Herzegovina	4.95	2.92	−41 (−57, −19)
Armenia	4.97	3.71	−25 (−47, 4)
Jordan	5.05	3.36	−34 (−50, −12)
Georgia	5.07	4.34	−14 (−35, 13)
Nigeria	5.29	3.64	−31 (−35, −27)
Cameroon	5.47	4.23	−23 (−33, −10)
South Sudan	6.00	14.00	139 (101, 185)
Albania	5.71	3.00	−47 (−63, −25)
Mongolia	5.78	5.19	−10 (−35, 25)
Myanmar	5.95	3.55	−40 (−45, −35)
The Gambia	6.11	4.34	−29 (−57, 16)
Belarus	6.25	4.40	−30 (−41, −16)
Kyrgyzstan	6.26	4.40	−30 (−45, −11)
Romania	6.42	4.50	−30 (−38, −21)
Turkmenistan	6.59	4.45	−32 (−47, −13)
Haiti	6.72	2.67	−60 (−68, −51)
Sri Lanka	6.79	3.74	−45 (−52, −37)
Cote d'Ivoire	6.81	4.25	−36 (−44, −27)
American Samoa	6.94	3.48	−50 (−95, 376)
Guinea−Bissau	6.96	3.75	−46 (−67, −13)
Estonia	7.02	2.86	−59 (−76, −30)
Senegal	7.04	4.71	−33 (−43, −21)
Uzbekistan	7.07	4.82	−32 (−39, −25)
Burkina Faso	7.15	2.89	−60 (−66, −52)
Nicaragua	7.27	4.77	−34 (−48, −18)
Guyana	7.54	5.33	−29 (−60, 24)
Liberia	7.72	5.44	−30 (−47, −6)

Lebanon	7.76	4.60	−41 (−54, −24)
Kenya	7.78	5.55	−29 (−35, −22)
Libya	7.82	4.67	−40 (−52, −26)
Philippines	8.12	4.29	−47 (−50, −44)
Ukraine	8.18	5.82	−29 (−34, −24)
Egypt	8.19	5.43	−34 (−37, −30)
Fiji	8.22	3.97	−52 (−73, −14)
Sierra Leone	8.34	4.92	−41 (−53, −26)
Colombia	8.38	3.09	−63 (−66, −60)
Kazakhstan	8.95	6.83	−24 (−31, −15)
Botswana	9.08	6.26	−31 (−51, −3)
Swaziland	9.27	3.71	−60 (−76, −33)
Malaysia	9.33	5.21	−44 (−49, −39)
Benin	9.34	5.48	−41 (−51, −30)
Honduras	9.37	10.61	13 (−4, 33)
Palestine	9.39	9.56	2 (−21, 30)
Maldives	9.48	3.73	−61 (−85, 3)
Panama	9.52	3.69	−61 (−71, −48)
Kiribati	9.58	5.17	−46 (−89, 155)
Marshall Islands	9.71	4.46	−54 (−94, 248)
Zambia	9.79	6.00	−39 (−47, −30)
Costa Rica	9.98	5.38	−46 (−57, −32)
North Korea	9.99	10.18	2 (−6, 10)
Samoa	10.02	3.49	−65 (−91, 28)
Djibouti	10.03	3.71	−63 (−80, −33)
Iran	10.04	5.65	−44 (−47, −41)
Tonga	10.17	4.55	−55 (−91, 115)
Lesotho	10.20	3.45	−66 (−77, −50)
Mexico	10.25	5.84	−43 (−45, −41)
Federated States of Micronesia	10.29	4.47	−57 (−91, 106)
Dominican Republic	10.31	8.47	−18 (−28, −6)
Solomon Islands	10.42	4.46	−57 (−80, −10)
Belize	10.73	7.87	−27 (−67, 65)
Togo	10.93	6.48	−41 (−51, −28)
Chad	11.54	6.54	−43 (−51, −34)
Brazil	11.55	7.24	−37 (−39, −35)
Comoros	11.56	5.84	−49 (−72, −9)
Pakistan	11.65	5.84	−50 (−52, −48)
Ecuador	11.83	9.25	−22 (−30, −13)
Russia	11.89	8.50	−28 (−31, −26)
Argentina	11.91	10.16	−15 (−20, −9)
Zimbabwe	11.93	5.29	−56 (−61, −50)
Timor−Leste	11.96	5.15	−57 (−73, −31)
Tunisia	12.00	7.51	−37 (−45, −29)
South Africa	12.40	7.35	−41 (−44, −37)
El Salvador	12.62	0.95	−93 (−95, −89)
Cuba	12.75	6.44	−49 (−55, −43)
Vietnam	12.89	5.32	−59 (−61, −57)
Syria	13.00	7.08	−46 (−51, −39)
Malawi	13.00	8.76	−33 (−40, −25)
China	13.27	7.51	−43 (−44, −43)
Mali	13.74	7.41	−46 (−52, −39)

Gabon	13.81	9.72	−30 (−50, −1)
Guinea	13.99	9.78	−30 (−39, −20)
India	14.31	5.58	−61 (−62, −61)
Paraguay	14.77	7.92	−46 (−54, −37)
Morocco	14.97	7.79	−48 (−51, −44)
Indonesia	15.31	13.29	−13 (−15, −11)
Bhutan	15.68	5.47	−65 (−79, −42)
Bangladesh	15.86	6.55	−59 (−60, −57)
Niger	16.06	12.35	−23 (−31, −15)
Algeria	16.51	11.54	−30 (−34, −26)
Laos	16.63	5.50	−67 (−72, −61)
Iraq	16.64	11.99	−28 (−33, −23)
Thailand	16.88	9.36	−45 (−47, −42)
Vanuatu	17.09	7.20	−58 (−82, −1)
Congo	17.10	11.02	−36 (−47, −22)
Uganda	17.56	7.61	−57 (−60, −53)
Tanzania	17.96	7.49	−58 (−61, −56)
Democratic Republic of the Congo	18.21	16.94	−7 (−11, −3)
Somalia	18.81	11.63	−38 (−45, −30)
Turkey	19.15	6.63	−65 (−67, −64)
Madagascar	19.16	10.16	−47 (−51, −42)
Papua New Guinea	20.89	7.95	−62 (−67, −56)
Cambodia	21.46	7.29	−66 (−69, −62)
Nepal	21.96	8.25	−62 (−65, −60)
Eritrea	22.27	7.71	−65 (−71, −59)
Burundi	22.43	12.28	−45 (−51, −39)
Mozambique	22.66	8.70	−62 (−65, −58)
Angola	23.67	12.03	−49 (−53, −45)
Guatemala	24.18	17.2	−29 (−34, −23)
Rwanda	24.86	7.62	−69 (−73, −66)
Sudan	25.42	9.62	−62 (−64, −60)
Equatorial Guinea	26.29	7.75	−71 (−81, −54)
Ethiopia	27.05	10.74	−60 (−62, −59)
Central African Republic	28.70	20.31	−29 (−38, −19)
Venezuela	30.71	24.42	−20 (−24, −17)
Yemen	43.41	11.56	−73 (−75, −72)
Afghanistan	61.16	25.96	−58 (−59, −56)

95% CI: 95% confidence interval; "—": 95% CI was unstable due to extremely small numbers of deaths and population.

Table S3. Country-specific unintentional occupational injury mortality (/100,000 persons) for age group 50–69 year in HICs between 1990 and 2016.

Country	1990	2016	% Change in Rates (95% CI)
United Kingdom	0.65	0.40	−39 (−56, −14)
Netherlands	0.92	0.77	−16 (−50, 39)
Trinidad and Tobago	1.24	0.69	−44 (−93, 329)
Norway	1.44	1.14	−21 (−64, 73)
Belgium	1.45	1.61	11 (−29, 74)
Sweden	1.47	0.93	−37 (−64, 12)
Denmark	1.55	1.41	−9 (−53, 75)
Finland	1.56	0.91	−42 (−72, 20)
Luxembourg	1.69	1.75	4 (—)
Malta	1.80	1.40	−22 (−93, 774)
Australia	2.03	1.27	−38 (−56, −12)
Andorra	2.27	1.55	−32 (—)
Ireland	2.45	1.70	−63 (−80, −32)
Slovenia	2.51	1.64	−35 (−73, 59)
New Zealand	2.57	2.31	−10 (−53, 71)
United States	2.59	2.29	−11 (−18, −5)
Germany	2.67	1.65	−38 (−46, −29)
Slovakia	2.73	1.63	−40 (−66, 5)
Greece	2.83	1.93	−32 (−52, −2)
Barbados	2.83	2.46	−13 (—)
France	2.96	3.47	17 (2, 34)
Hungary	3.07	1.33	−57 (−71, −35)
Bermuda	3.13	2.69	−14 (—)
Iceland	3.23	2.01	−38 (—)
Switzerland	3.24	2.49	−23 (−48, 14)
Austria	3.37	1.79	−47 (−65, −20)
The Bahamas	3.49	2.02	−42 (—)
Czech Republic	3.62	2.75	−24 (−45, 4)
Israel	3.67	1.37	−63 (−80, −32)
Cyprus	3.84	2.61	−32 (−81, 143)
Antigua and Barbuda	3.89	2.27	−42 (—)
Latvia	4.22	3.94	−7 (−48, 68)
Italy	4.24	2.63	−38 (−45, −30)
Lithuania	4.32	3.32	−23 (−54, 29)
Canada	4.58	3.28	−28 (−40, −15)
Portugal	4.62	2.72	−41 (−56, −20)
Poland	4.63	2.03	−56 (−63, −48)
Uruguay	4.96	5.66	14 (−29, 84)
Greenland	5.10	3.65	−28 (—)
Puerto Rico	5.17	3.07	−41 (−65, 2)
Chile	5.47	5.70	4 (−18, 33)
Spain	5.48	1.29	−76 (−80, −72)
Guam	5.83	1.85	−68 (—)
Seychelles	7.76	3.29	−58 (—)
Taiwan	8.14	3.74	−54 (−62, −45)
Bahrain	8.50	6.12	−28 (−80, 164)
Singapore	8.78	3.09	−65 (−79, −42)
Japan	9.86	2.98	−70 (−72, −68)
Virgin Islands, U.S.	10.41	7.35	−29 (−91, 446)

Kuwait	13.16	7.33	−44 (−68, −2)
Qatar	15.83	11.38	−28 (−73, 91)
United Arab Emirates	16.79	11.56	−31 (−59, 17)
Saudi Arabia	19.27	7.26	−62 (−69, −55)
South Korea	20.91	3.98	−81 (−83, −79)
Oman	27.47	8.17	−70 (−82, −52)
Brunei	29.32	17.59	−40 (−78, 67)

95% CI: 95% confidence interval; "—": 95% CI was unstable due to extremely small numbers of deaths and population.

Table S4. Country−specific unintentional occupational injury mortality (/100,000 persons) for age group 50−69 year in LMICs between 1990 and 2016.

Country	1990	2016	% Change in Rates (95% CI)
Saint Vincent and the Grenadines	0.56	0.39	−30 (—)
Bulgaria	0.84	2.25	169 (56, 364)
Croatia	1.41	1.12	−21 (−62, 65)
Mauritius	1.94	0.75	−61 (−94, 142)
Montenegro	2.41	1.51	−37 (−89, 263)
Macedonia	2.87	2.16	−25 (−68, 78)
Serbia	2.98	2.11	−29 (−51, 3)
Bosnia and Herzegovina	3.36	1.97	−41 (−67, 3)
Mauritania	3.73	2.82	−25 (−72, 102)
Jordan	3.79	2.24	−41 (−73, 31)
Moldova	3.91	3.59	−8 (−43, 49)
Dominica	4.04	1.88	−54 (—)
Sao Tome and Principe	4.19	3.07	−27 (—)
Azerbaijan	4.24	3.69	−13 (−41, 28)
Cape Verde	4.42	2.44	−45 (−95, 469)
Belarus	4.44	3.15	−29 (−47, −5)
Namibia	4.72	3.01	−36 (−79, 96)
Grenada	4.78	1.99	−58 (—)
Saint Lucia	4.78	1.66	−65 (—)
Nigeria	4.89	3.45	−29 (−38, −20)
Ghana	4.95	3.63	−27 (−47, 2)
Romania	4.96	2.87	−42 (−53, −29)
Georgia	4.98	4.48	−10 (−40, 34)
Estonia	4.99	1.98	−60 (−84, −3)
Armenia	5.23	3.71	−29 (−58, 19)
Suriname	5.38	3.67	−32 (−87, 248)
Albania	5.50	2.26	−59 (−79, −21)
Bolivia	5.70	4.86	−15 (−43, 28)
Egypt	5.81	4.07	−30 (−39, −20)
Jamaica	5.82	3.53	−39 (−70, 23)
Cameroon	5.83	4.48	−23 (−45, 7)
Kyrgyzstan	5.89	3.67	−38 (−62, 2)
Peru	6.06	4.83	−20 (−36, −1)
Libya	6.24	3.74	−40 (−65, 3)
Palestine	6.30	9.00	43 (−33, 202)
Ukraine	6.53	3.52	−46 (−52, −39)
American Samoa	6.72	3.16	−53 (—)
Burkina Faso	6.75	2.72	−60 (−74, −38)
Guinea−Bissau	6.76	3.89	−43 (−81, 70)
Turkmenistan	6.94	4.22	−39 (−64, 3)
Lebanon	7.16	4.85	−32 (−58, 9)
Mongolia	7.16	3.79	−47 (−74, 9)
Cote d'Ivoire	7.17	4.88	−32 (−50, −8)
Fiji	7.20	2.94	−59 (−89, 51)
Myanmar	7.28	3.71	−49 (−56, −40)
Senegal	7.34	4.50	−39 (−59, −9)
The Gambia	7.40	5.02	−32 (−79, 124)
Colombia	7.46	2.42	−68 (−73, −61)
Uzbekistan	7.53	4.62	−39 (−50, −24)

Tajikistan	7.64	5.89	−23 (−49, 16)
Sri Lanka	7.77	3.31	−57 (−66, −47)
Liberia	7.89	5.40	−32 (−65, 33)
Costa Rica	7.97	4.76	−40 (−63, −3)
Sierra Leone	8.03	4.76	−41 (−65, 0)
Iran	8.27	4.29	−48 (−55, −41)
Nicaragua	8.45	4.72	−44 (−66, −8)
Tunisia	8.48	5.96	−30 (−47, −7)
Kiribati	8.54	3.82	−55 (—)
Tonga	8.71	4.09	−53 (—)
Cuba	8.77	10.21	16 (−5, 43)
Panama	9.05	2.83	−69 (−83, −42)
Samoa	9.08	3.07	−66 (−98, 464)
Mexico	9.36	5.25	−44 (−49, −38)
Russia	9.62	5.60	−42 (−45, −39)
Federated States of Micronesia	9.63	3.64	−62 (—)
Haiti	9.68	3.64	−62 (−74, −45)
Guyana	9.80	6.78	−31 (−75, 91)
Kazakhstan	9.87	5.90	−40 (−51, −28)
Marshall Islands	9.97	4.10	−59 (—)
Vietnam	9.97	4.62	−54 (−58, −49)
Honduras	10.18	14.32	41 (−2, 102)
Benin	10.20	6.23	−39 (−59, −9)
Lesotho	10.50	2.97	−72 (−89, −24)
Philippines	10.56	4.61	−56 (−61, −51)
Botswana	10.56	6.44	−39 (−72, 34)
Brazil	10.59	5.68	−46 (−50, −43)
Argentina	10.84	8.99	−17 (−26, −7)
El Salvador	11.07	0.83	−92 (−96, −84)
Togo	11.07	6.54	−41 (−63, −6)
Morocco	11.15	6.41	−42 (−51, −33)
Zambia	11.28	6.96	−38 (−56, −14)
Solomon Islands	11.31	4.54	−60 (−93, 131)
Chad	11.67	6.86	−41 (−59, −16)
Malaysia	11.72	4.56	−61 (−68, −53)
Belize	11.78	7.43	−37 (−90, 306)
Swaziland	11.88	3.86	−68 (−90, 7)
North Korea	11.96	10.01	−16 (−27, −4)
Syria	11.98	6.61	−45 (−57, −29)
South Africa	12.25	8.10	−34 (−42, −25)
Pakistan	12.30	5.57	−55 (−58, −51)
Timor−Leste	12.67	5.29	−58 (−86, 26)
Maldives	12.67	3.69	−71 (−96, 107)
Dominican Republic	13.09	8.85	−32 (−48, −12)
Comoros	13.10	6.28	−52 (−87, 82)
India	13.42	5.14	−62 (−63, −61)
Iraq	13.56	9.42	−31 (−42, −16)
Djibouti	13.78	4.63	−66 (−90, 13)
Algeria	13.89	8.70	−37 (−46, −27)
Gabon	14.12	10.46	−26 (−62, 44)
Zimbabwe	14.13	5.63	−60 (−71, −45)
China	14.79	6.57	−56 (−56, −55)

Mali	14.85	7.37	−50 (−62, −34)
Turkey	15.49	4.16	−73 (−76, −70)
Paraguay	15.53	7.57	−51 (−66, −31)
Ecuador	15.53	9.64	−38 (−50, −24)
Kenya	15.93	7.23	−55 (−62, −46)
Bhutan	16.63	6.00	−64 (−88, 7)
Guinea	16.99	10.24	−40 (−54, −21)
Niger	17.86	14.27	−20 (−36, 0)
Bangladesh	18.02	6.65	−63 (−66, −60)
Thailand	19.32	8.24	−57 (−60, −54)
Vanuatu	19.73	7.62	−61 (−94, 146)
Congo	19.95	12.63	−37 (−58, −5)
Sudan	20.15	8.45	−58 (−64, −51)
Malawi	20.56	8.42	−59 (−68, −47)
Tanzania	20.92	8.10	−61 (−67, −55)
Papua New Guinea	20.93	7.01	−67 (−77, −52)
Madagascar	21.09	10.21	−52 (−60, −41)
Laos	22.67	6.75	−70 (−79, −58)
Nepal	23.69	8.10	−66 (−71, −60)
Cambodia	24.35	6.95	−71 (−77, −64)
Democratic Republic of the Congo	24.37	22.10	−9 (−17, −1)
Burundi	24.56	13.69	−44 (−57, −27)
Uganda	25.79	8.51	−67 (−72, −61)
Equatorial Guinea	26.46	9.64	−64 (−85, −11)
Somalia	27.27	12.69	−53 (−64, −40)
Eritrea	27.35	7.85	−71 (−82, −55)
Indonesia	27.59	18.65	−32 (−35, −30)
Guatemala	29.19	15.85	−46 (−55, −35)
Angola	31.38	14.08	−55 (−62, −47)
Mozambique	31.42	9.72	−69 (−74, −63)
Yemen	32.12	9.45	−71 (−76, −65)
Venezuela	32.51	22.17	−32 (−38, −25)
Central African Republic	33.07	22.08	−33 (−50, −12)
Rwanda	34.36	8.08	−76 (−82, −69)
South Sudan	34.98	24.53	−30 (−42, −15)
Ethiopia	38.53	10.33	−73 (−75, −71)
Afghanistan	48.24	24.67	−49 (−55, −42)

95% CI: 95% confidence interval; "—": 95% CI was unstable due to extremely small numbers of deaths and population.
